# Restoration of Corticosteroid Sensitivity by p38 Mitogen Activated Protein Kinase Inhibition in Peripheral Blood Mononuclear Cells from Severe Asthma

**DOI:** 10.1371/journal.pone.0041582

**Published:** 2012-07-23

**Authors:** Nicolas Mercado, Amir Hakim, Yoshiki Kobayashi, Sally Meah, Omar S. Usmani, Kian Fan Chung, Peter J. Barnes, Kazuhiro Ito

**Affiliations:** 1 Airway Disease Section, National Heart and Lung Institute, Imperial College, London, United Kingdom; 2 Biomedical Research Unit, Royal Brompton Hospital and Imperial College, London, United Kingdom; Harvard School of Public Health, United States of America

## Abstract

**Background:**

Severe asthma accounts for a small number of asthmatics but represents a disproportionate cost to health care systems. The underlying mechanism in severe asthma remains unknown but several mechanisms are likely to be involved because of a very heterogeneous profile. We investigated the effects of a p38MAPK inhibitor in corticosteroid sensitivity in peripheral blood mononuclear cells (PBMCs) from severe asthmatics and the profile of its responders.

**Methodology/Principal Findings:**

Corticosteroid sensitivity was determined by measuring dexamethasone inhibition of CD3/28 and TNF-α induced IL-8 production in PBMCs by using ELISA. PBMCs from severe asthmatics were relatively less sensitive to dexamethasone (Dex) as compared to those of non-severe asthmatics and healthy volunteers. The IC_50_ values of Dex negatively correlated with decreased glucocorticoid receptor (GR) nuclear translocation assessed using immunocytochemistry (r = −0.65; p<0.0005) and with decreased FEV_1_ (% predicted) (r = 0.6; p<0.0005). A p38α/β inhibitor (SB203580) restored Dex-sensitivity in a subpopulation of severe asthma that was characterized by a defective GR nuclear translocation, clinically by lower FEV_1_ and higher use of oral prednisolone. We also found that SB203580 partially inhibited GR phosphorylation at serine 226, resulting in increased GR nuclear translocation in IL-2/IL-4 treated corticosteroid insensitive U937s.

**Conclusions/Significance:**

p38MAPKα/β is involved in defective GR nuclear translocation due to phosphorylation at Ser226 and this will be a useful biomarker to identify responders to p38MAPKα/β inhibitor in the future.

## Introduction

Most patients with asthma have mild to moderate forms of the disease and are well controlled by corticosteroids or a combination of corticosteroids and long-acting β_2_-adrenoreceptors agonists (LABA). However, between 5–10% of patients remain symptomatic despite treatment with high doses of corticosteroids [1], [2]. This group of patients account for about 50% of total health care cost in asthma [3]. It remains unclear as to why these patients respond less to inhaled and oral corticosteroids. Therefore, it is important to investigate both clinical and molecular features of corticosteroid resistance in severe asthma in order to better understand the complexity of the disease and identify any specific treatment.

It is widely acknowledged that heterogeneous mechanisms are involved in corticosteroid insensitivity. Lymphocytes and monocytes have been shown to be less corticosteroid sensitive in severe asthmatics as compared to non-severe form [4], [5]. Increased IL-2, IL-4 in bronchoalveolar lavage (BAL) cells and IL-13 in sputum and lung biopsies have been observed in severe asthmatics and these cytokines are known to cause *in vitro* loss of corticosteroid responsiveness [5]–[7]. An increase in the inactive GRβ isoform and a decrease in nuclear translocation have also been identified as causes of corticosteroid insensitivity in severe asthma [8], [9]. The phosphorylation status of GR is reported to play a crucial role in its function and localization [5], [10]. Additionally, activated pro-inflammatory transcription factors NF-κB and AP-1 can sequester GR or compete for transcription co-factors [1], [11]. Smoking asthmatics have also shown reduced systemic corticosteroid responsiveness and oxidative stress could affect histone deacetylase (HDAC) 2 level which is critical for the mechanism of GR trans-repression [12], [13].

Standard treatment for corticosteroid insensitive severe asthma includes high doses of inhaled corticosteroid combined with LABA [14] as well as the use of leukotriene receptor antagonists, anticholinergics or theophylline [15]. Systemic corticosteroids are needed in patients with severe unremitting disease although the risk of side effects is significantly increased [15]. Other therapies have been investigated with mixed results including immunosuppressants anti-IgE and anti-TNFα [16]. The search for new therapies is particularly directed towards add-ons treatment to corticosteroid that can overcome the decrease in sensitivity observed in severe asthmatics. Inhibitors to kinases (p38, JNK, ERK, PI3K) and pro-inflammatory transcription factors (AP-1, NF-κB) are of particular interest. The p38MAPK pathway regulates various pro-inflammatory transcription factors such as AP-1 and NF-κB [17], [18]. p38MAPK activation can also stabilize pro-inflammatory cytokines and chemokines transcripts [19] but also lead to the phosphorylation and inactivation of the glucocorticoid receptor (GR) and subsequent corticosteroid insensitivity [5]. This is supported by recent evidence that confirmed an increase in p38MAPK activation in alveolar macrophages from severe asthmatics [20]. In addition, the p38MAPK inhibitor, SB681323, also inhibited cytokine production in blood stimulated *ex vivo* in COPD patients [21].

In the present study we showed that a p38MAPK inhibitor (SB203580) preferentially restored corticosteroid sensitivity in PBMCs from a subpopulation of severe asthma that were characterized by increased *ex-vivo* corticosteroid insensitivity, decreased GR nuclear translocation and clinically by a tendency for reduced lung function and higher use of oral corticosteroids.

## Results

### Corticosteroid Sensitivity to IL-8 was Reduced in Severe Asthma

**Figure 1:**
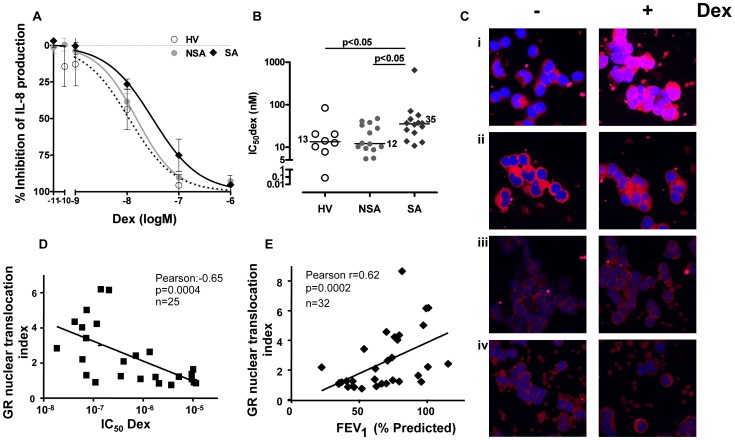
Corticosteroid sensitivity and GR nuclear translocation in asthma. A. Dex (10^−11^–10^−6^ M) was incubated 1 hour followed by 24 hours stimulation with TNFα. ELISA was used to measure IL-8 levels in 8 healthy volunteers (HV), 14 non-severe asthmatics (NSA), and 14 severe asthmatics (SA). IC_50_dex (50% inhibitory concentration) was measured and plotted in graph **B. B.** IC_50_dex measured from **A.** was plotted for 8 HV, 14 NSA and 14 SA. **C.** Example of nuclear translocation as assessed by immunocytochemistry of PBMCs treated with Dex (1 µM) for 4 hrs. PBMCs were cytospined into slides and air dried. GR was detected using an anti-GR antibody with a secondary cy3-conjugated antibody (red). The nucleus was counter-stained using a cy5 To-Pro-3 (blue). A fixed area was drawn and used to measure the intensities of the red and blue channels in the nucleus. Ten cells per experiment were counted and the ratio cy3/cy5 used as the representation of nuclear GR which was normalized for Dex treatment. The fold induction of the signal ratio of 4 hours incubation with Dex over non-treatment was calculated as the index of GR nuclear translocation (GNI: GR nuclear translocation index). Four patients’ pictures from confocal microscopy are shown. (−); non-treatment, (Dex); Dex (1 µM) for 4 hrs. (i) Healthy volunteer with GNI = 4.2. (ii) Non-severe asthmatic with GNI = 2.4. (iii) Severe asthmatic with GNI = 1.2. (iv) Severe asthmatic with GNI = 1.2. **D.** Correlation between GNI and IC_50_dex in all asthmatics (n = 24). **E.** Correlation between GNI and lung function as measured by FEV_1_ (%pred) in asthma (n = 32). Data was plotted as median ± SEM. *p*<0.05 is significant.

Ten healthy subjects, 20 patients with mid-to-moderate asthma and 20 patients with severe asthma have been recruited for this study (Table 1). Basal levels of IL-8 in PBMCs after overnight incubation were similar between patient groups (Table 2). TNFα stimulation alone resulted in a 6 to 8 fold increase of IL-8 production in all patients with no significant differences between groups (Table 2). However, concentration-dependent inhibition curve of dexamethasone on TNFα-induced IL-8 shifted to the right in severe asthma compared with those of healthy volunteers and non-severe asthma patients, resulting in higher 50% inhibitory-concentration of dexamethasone (IC_50_dex) for severe asthmatics (median (range): 35.4 (19.8;48.4), n = 14; p<0.05) as compared to non-severe asthmatics (12.2 (9.2;34.3) nM, n = 14) and healthy volunteers (13.4 (8.4;20.3) nM, n = 8) (Figures 1A and 1B).

**Table 1:** Patients’ characteristic.

	*Healthy Volunteer*	*Non-Severe Asthma*	*Severe Asthma*
Number	10	20	20
Gender (M/F)	3/7	10/10	6/14
Age	32 (30;40)	43 (31;52)	43 (35;60)
Atopy	0/10	18/20	17/20
FEV_1_ (% pred)*	102 (88;108)	80 (75;95)	80 (41;73)||§
FEV/FVC†	97 (95;103)	92 (79;100)	80 (71;93)
			(n = 19)**
PEF (L/M)‡	494 (391;542)	374 (303;564)	236 (162;310)
	(n = 9)		(n = 19)||**
Beclamethasone equivalent (ug)	0	75 (0;400)	1000 (800;2000)††
Prednisolone (mg)	0	0	10.0 (1.3;18.8)††
Smoking	0	0	1

Values are expressed median (interquartile range) except for gender, Atopy, smoking status.

* FEV_1_: Forced expiratory volume in one second.

† FVC: Forced vital capacity.

‡ PEF: Peak expiratory flow.

** p<0.01 compare to HV.

|| p<0.0001 compare to HV.

§ p<0.001 compare to NSA.

†† p<0.0001 compare to HV. and NSA.

**Table 2:** Molecular profile of healthy volunteers, non-severe and severe asthmatics.

	*Healthy Volunteer*	*Non-Severe Asthma*	*Severe Asthma*
Number	10	20	20
Basal IL-2 (pg/ml)	0.0 (0.0;2.6)	0.6 (0.0;7.3)	5.6 (0.0;19.0)**
Basal IL-4 (pg/ml)	0.0 (0.0;0.0)	0.0 (0.0;0.5)	3.5 (0.0;4.9)‡
Basal IL-8 (pg/ml)	438 (162;1174)	412 (163;854)	207 (77;494)
TNFα-induced IL-8 (pg/ml)	2642 (822;4070)	3200 (2175;3891)	2140 (1233;3485)
	(n = 8)	(n = 14)	(n = 14)
CD3/28+ TNFα-induced IL-8 (pg/ml)	8277 (6919;9270)	7864 (5163;8910)	9102 (7662;11228)
GRα protein (β-actin normaliz.)	0.05 (0.01;0.09)	0.04 (0.02;0.12)	0.05 (0.02;0.08)
	(n = 8)	(n = 14)	(n = 18)
HDAC2 protein (β-actin normaliz.)	0.08 (0.05;0.30)	0.12 (0.08;0.25)	0.07 (0.02;0.14)§
	(n = 8)		(n = 18)
IC_50_dex of IL-8 (TNFα) (nM)	13.4 (8.4;20.3)	12.2 (9.2;34.3)	35.4 (19.8;48.4)††
	(n = 8)	(n = 14)	
Ratio of **CI/CS** * (TNFα/anti-CD3/28)	1/10	2/20	6/20
GR nuclear translocation index (Dex/NT)†	2.2 (1.8;3.2)	2.4 (1.3;4.8)	1.3 (1.1;2.6)§
	(n = 9	(n = 13)	(n = 19)

Values are expressed median (interquartile range) except for ratio of CI/CS.

* CI (steroid insensitive; IC_50_dex>10^−4^ M) and CS (steroid sensitive IC_50_dex<10^−4^ M) as measured after anti-CD3/28/TNFα stimulation.

† Dex: dexamethasone, NT: Negative control.

** p<0.001 compared to HV.

‡ p<0.01 compared to HV and NSA.

§ p<0.05 compared to NSA.

†† p<0.05 compared to HV and NSA.

When the co-stimulation of TNFα with anti-CD3/28 was used in order to stimulate lymphocytes as well as monocytes, the production of IL-8 increased by 3–5 fold compared to TNFα alone, and the levels were not different between groups (Table 2). This system was less sensitive to dexamethasone as IC_50_dex was higher, and PBMCs from some patients did not inhibit IL-8 production in the range of dexamethasone concentrations used. In that case, “10^−4^ M” was arbitrarily used as IC_50_dex as maximal quantifiable data. The IC_50_dex of anti-CD3/28 and TNFα-induced IL-8 in PBMCs from healthy volunteers was 1.26 (median) (0.46;7.74) µM (n = 10), which was more than 100 times higher than IC_50_dex of TNFα-induced IL-8 production alone. IC_50_dex value of non-severe asthmatics was 0.09 (0.02;4.35) µM (n = 20) (Fig. S1A), significantly lower than theIC_50_dex value of severe asthmatics (2.87 (0.19;100) µM). The percentage of patients that did not response to dexamethasone up to 10^−5^ M was 33% in severe asthma, which was higher than in healthy volunteers and non-severe asthmatics (10% each) (Table 2).

Basal IL-2 level in PBMCs from patients with severe asthma (median (range): 5.6 (0.0;19.0) pg/ml, n = 10; p<0.05) was significantly higher compared to those of healthy volunteers (0.0 (0.0;2.6) pg/ml, n = 10). IL-2 levels from non-severe asthmatics (0.6 (0.0;7.3) pg/ml, n = 10) were high in a couple of cases but not significantly different from those in any other groups (Fig. S1B). As IL-2 and IL-4 have been reported to induce corticosteroid insensitivity, the basal levels of IL-4 were also determined and found to be increased in severe asthmatics (3.5 (0.0;4.9) pg/ml, n = 20) compared to healthy volunteers (0.0 (0.0;0.0) pg/ml, n = 10; p<0.01) and non-severe asthmatics (0.0 (0.0;0.5) pg/ml, n = 20; p<0.01) (Figure S1C).

### Impaired GR Nuclear Translocation is Associated with Loss of Corticosteroid Sensitivity and Disease Severity

GR nuclear translocation was determined as the ratio of mean fluorescence between the GR signals (cy3 channel; red) in a fixed area of the nuclei and the nuclear signal (cy5 channel; blue) of the same area (Figure 1C). The fold induction of the signal ratio of 4 hours dexamethasone treatment over non-treatment was calculated as the index of GR nuclear translocation (GNI: GR nuclear translocation index). The antibody used for immunocytochemistry is specific only for GRα isoform. Ten cells were randomly selected in each slide and the average GNI was calculated (Fig. S1D). Figure 1C showed representative results of the PBMCs from four individuals treated with or without dexamethasone: one healthy volunteer (i; GNI = 4.2), one non-severe asthmatic (ii; GNI = 2.4) and two severe asthmatics (iii; GNI = 1.2 and iv; GNI = 1.2). This semi-quantitative analysis demonstrated that the GNI in severe asthma (1.3 (1.1;2.6) ratio, n = 19) tended to be lower when compared with GNIs of healthy volunteers (2.2 (1.8;3.2), n = 9; p = 0.18) but significantly decreased when compared to non-severe asthmatics (2.4 (1.3;4.8), n = 14; p<0.05) (Fig. S1D). When comparing the IC_50_dex for IL-8 and the GNI in patients, there was a strong correlation (r = −0.65, p<0.0005, n = 25; Figure 1D), suggesting less GR nuclear translocation was associated with less inhibitory efficacy of dexamethasone on cytokine release. Furthermore, there was also a good correlation between FEV_1_ (% predicted) and GNI in asthmatics (r = 0.62, p<0.0005, n = 32), suggesting that patients showing a defective GR nuclear translocation were more severe and more corticosteroid insensitive. No differences in GRα expression were observed between patient groups (Table 2). GRβ was not observed using this antibody which can detect both isoforms (data not shown).

Western Blot analysis demonstrated a small but significant reduction in HDAC2 expression in severe asthmatics (ratio of band densities of HDAC2 and Lamin A/C: 0.07 (0.02;0.14), n = 18) compared to non-severe asthmatics only (0.12 (0.08;0.25); n = 20; p<0.05) (Fig. S1D). However, HDAC2 expression levels were not associated with lung function or dexamethasone sensitivity in these samples.

### p38MAPK Inhibition Restores Corticosteroid Sensitivity in Severe Asthma

**Figure 2:**
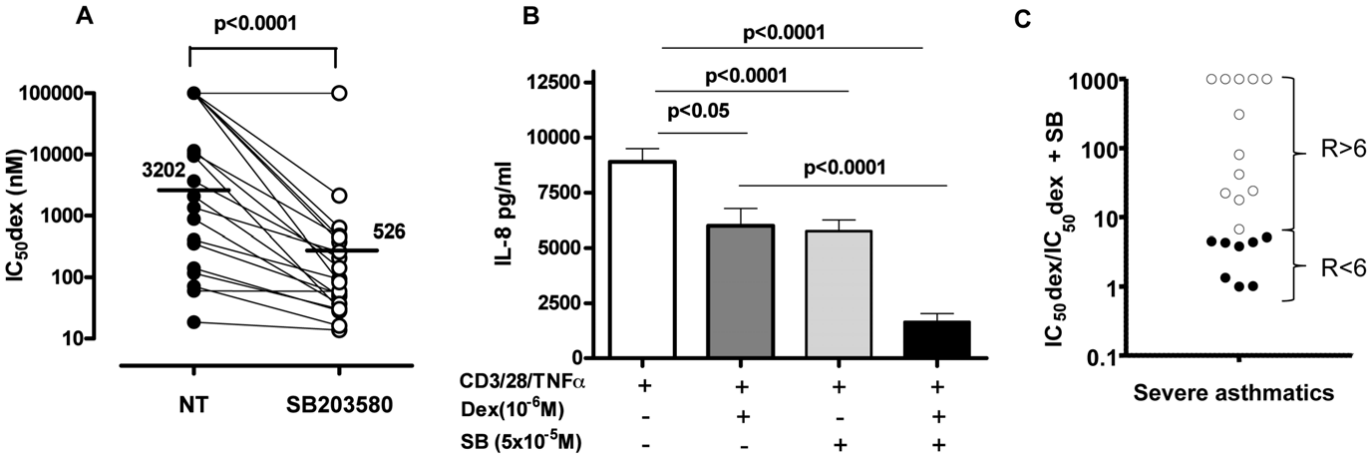
p38MAPK inhibition restores corticosteroid sensitivity in severe asthma. **A.** PBMCs from severe asthmatics were incubated 30 minutes with SB203580 (5 µM) and followed by treatment with Dex (10^−11^–10^−6^ M) for 1 hour and 24 hour with anti-CD3/28 plus TNFα. IL-8 release was measured by ELISA and IC_50_dexs were calculated with or without SB203580 (n = 20). **B.** PBMCs from severe asthmatics were treated with anti-CD3/28 plus TNFα and IL-8 concentrations calculated in pg/ml. Cells were pre-incubated with Dex (1 µM) alone or in combination with SB203580 (5 µM) prior anti-CD3/28 plus TNFα stimulation (n = 20). Data was plotted as median ± SEM. *p*<0.05 is significant. **C.** The improvement on corticosteroid sensitivity was assessed for SB203580 by calculating the ratio (fold) change of IC_50_dex before and after treatment. Some patients did not show inhibition in the range of Dex concentrations used. In that case, “10^−4^ M” was arbitrarily used as IC_50_dex as maximal quantifiable data. When SB203580 incubation restored a response to Dex in Dex-insensitive patients, the improvement was arbitrarily designated as 1000 fold. Patients were divided into those who respond more to SB203580 (ratio >6, white dots, n = 12) than those who respond less (ratio <6, black dots, n = 8).

A p38MAPKα/β inhibitor (SB203580), was incubated for 30 minutes prior to dexamethasone treatment and the individual changes in corticosteroid sensitivity (IC_50_dex without treatment/IC_50_dex with treatment) were determined in each individual with severe asthma (Fig. S2A). SB203580 increased corticosteroid sensitivity more or less in all severe asthma patients (Figure 2A), and particularly the patients showed more than 6 improvement index (ratio of IC_50_dex with and without treatment) by SB203580 in 12 out of 20 severe asthmatics (Figure 2C). In these samples formoterol (1 µM) did not improve corticosteroid sensitivity (Fig. S2A). SB203580 alone inhibited TNFα and CD3/CD28 induced IL-8 production in severe asthma (SB: 5978±506 pg/ml vs. NT: 9102±606 pg/ml; p<0.0001) (Figure 2B) but the maximum inhibition of dexamethasone (10^−6^ M) on CD3/28 and TNFα-stimulation of IL-8 was limited (Dex (10^−6^ M): 7421±779 pg/ml vs. NT: 9102±606 pg/ml; p<0.05). However, combination of SB203580 and dexamethasone resulted in a stronger inhibition of IL-8 compared to Dex (DEX +SB: 1163±369 pg/ml; p<0.0001 vs Dex (10^−6^ M), Figure 2B) or SB203580 alone.

### p38MAPK Inhibition Restored Corticosteroid Sensitivity and Reduced GR Serine-226 Phosphorylation in Corticosteroid-resistant *in vitro* U937 Model

**Figure 3:**
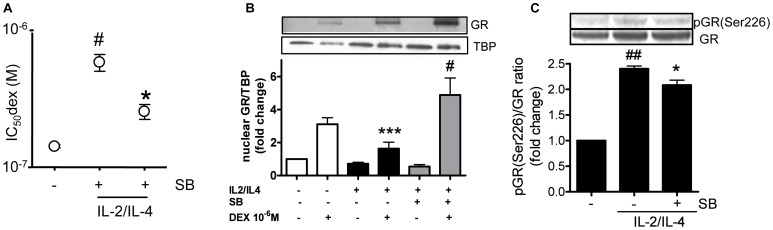
Effect of SB203580 on steroid sensitivity and GR nuclear translocation in U937. A. U937 cells were initially incubated with IL-2/IL-4 for 48 hours. Cells were pre-treated with SB203580 (5 µM) for 30 min followed by Dex (10^−11^−10^−6^ M) for 1 hour and TNFα stimulation (10 ng/ml) overnight. TNFα-induced IL-8 release was evaluated by ELISA and IC_50_dex values for Dex on IL-8 production were calculated. Values represent means of three experiments ± SEM. # *p*<0.05 (vs. non-treatment control; NT), and* *p*<0.01 (vs. treatment with IL-2/IL-4 only). **B.** U937 cells were incubated with IL-2/IL-4 for 48 hours. Cells were then stimulated with SB203580 (5 µM) for 30 min followed by Dex 10^−6^ M for 4 hours. Nuclear protein was extracted and GR was detected using SDS-PAGE/Western Blotting. TBP was detected as loading control. Ratio of GR nuclear translocation was calculated dividing GR absorbance by TBP.*** *p*<0.001 (IL-2/IL-4+ dex vs. treatment with IL-2/IL-4 only), # *p*<0.05 (IL-2/IL-4+ dex + SB vs. treatment with IL-2/IL-4+ dex), n = 3. **C.** U937s were stimulated with IL-2/4 for 48 hours and then stimulated with SB203580 (5 µM) for 30 minutes prior whole-cell extraction and SDS-PAGE/Western-Blotting. Phosphorylation of Serine 226 was determined with anti-S226 GR antibody normalized to GR expression. The band density was calculated by densitometry. ## *p*<0.01 (vs. non-treatment control),* *p*<0.05 (vs. treatment with IL-2/IL-4 only), n = 4.

IL-2 and IL-4 were increased in severe asthmatics (Figs. S1B and S1C), suggesting a possible role in reduced corticosteroid sensitivity. Treatment of U937s with IL-2/4 for 48 hours reduced corticosteroid sensitivity on TNFα-induced IL-8 production (increased IC_50_dex: IL-2/4: 602.6±86.4 nM vs. NT: 143.1.6±6.2 nM; p<0.01). Pre-incubation with SB203580 partially restored corticosteroid sensitivity (IC_50_dex: SB: 317.3±33.1 nM vs. IL-2/4; p<0.05). IL-2/4 pre-treatment also reduced dexamethasone induced GR nuclear translocation as observed in Figure 3B (fold induction of nuclear translocation over NT: NT + dex  = 3.1 vs. IL-2/4+ dex  = 1.6; p<0.001) and SB203580 significantly increased dexamethasone induced GR translocation (fold induction of nuclear translocation: IL-2/4+ dex + SB = 4.9 vs. IL-2/4+ dex  = 1.6; p<0.05). In addition, GR phosphorylation at Ser226 was clearly phosphorylated with IL-2/IL-4 treatment, and it was partially but significantly inhibited by SB203580 (Figure 3C).

### Characteristics of p38MAPK Inhibitor Responders in Severe Asthma

**Figure 4:**
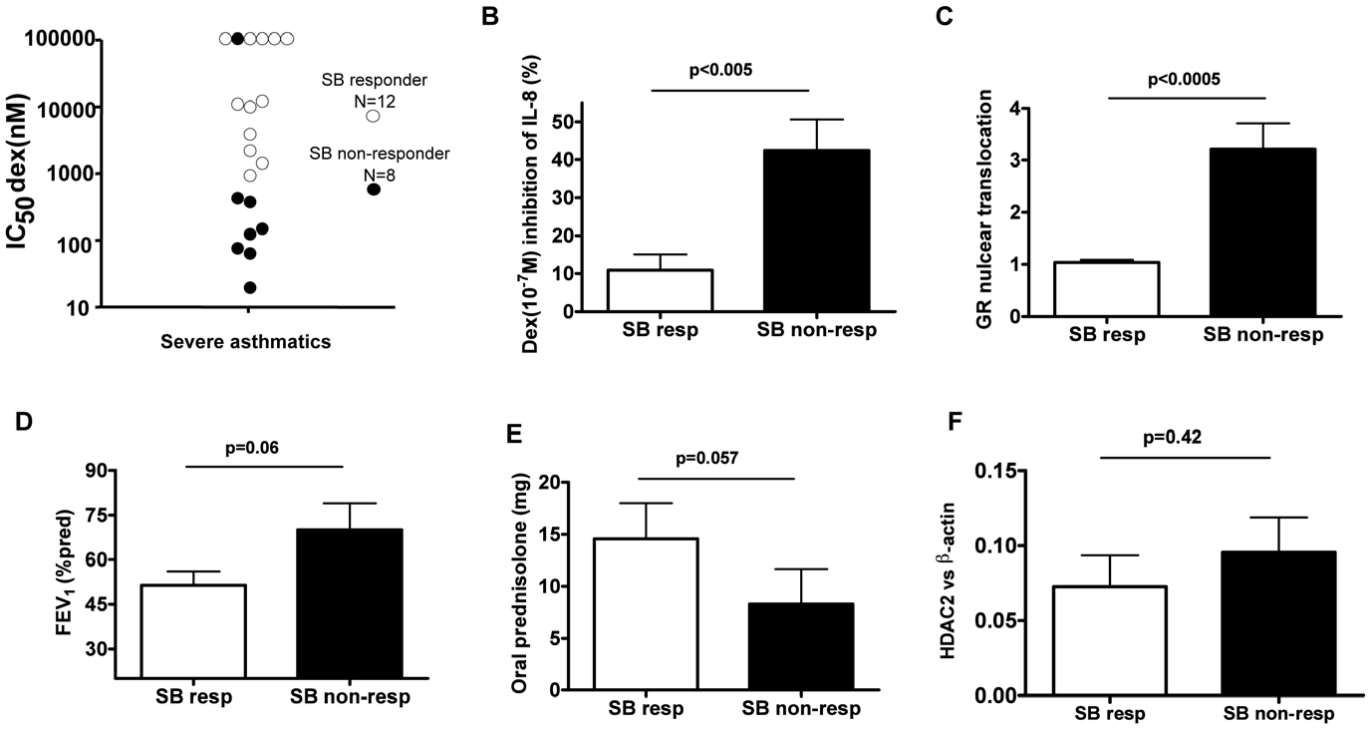
Two populations in severe asthma based on response to SB203580. Severe asthmatics were arbitrarily separated into SB203580 “responders” or “non-responders” based on the ratio IC_50_dex without treatment/IC_50_dex with treatment >6 (SB resp, n = 12) or ratio <6 (SB non-resp, n = 8) and compared using various parameters: **A.** IC_50_dex for IL-8. **B.** The % inhibition of anti-CD3/28 and TNFα-induced IL-8 after dex (10^−7^ M) treatment. **C.** Ratio of GR nuclear translocation dex (1 µM)/NT. **D.** Lung function by FEV_1_ (% predicted). **E.** Use of oral prednisilone (mg). **F.** HDAC2 protein expression normalized with β-actin. Data was plotted as mean ± SEM. *p*<0.05 is significant.

In Figure 2C, the effect of SB203580 on restoration of corticosteroid sensitivity varied greatly in severe asthmatics. In order to characterize why some patients responded better than others we arbitrarily divided severe asthmatics into SB203580 “*in vitro* higher-responders” (SB-higher-responders; improvement index, IC_50_dex without treatment/IC_50_dex with treatment >6; n = 12) and “low/non-responders” (SB-low/non-responders; improvement index<6; n = 8) (Figure 2B). Figure 4A showed that SB-higher responders were more corticosteroid insensitive when comparing IC_50_dex of SB-low/non responders. Likewise, the inhibitory effects of dexamethasone (10^−7^ M) on IL-8 release was significantly lower in SB-higher responders than SB-low/non responders (SB-higher responders: 10.9±4.2% vs. SB-low/non responders: 42.4±8.3%; p<0.005, Figure 4B). SB-higher responders also had a defect of GR nuclear translocation (GNI: SB-higher responders: 1.04±0.05 vs. SB-low/non responders: 3.21±0.50; p<0.0005, Figure 4C). SB-higher responders moreover showed a tendency for lower lung function score (FEV_1_ (% pred) (p = 0.06), Figure 4D, FEV/FVC (% pred) (p = 0.22) and PEF L/M (p = 0.14)) and use of higher doses of oral prednisolone (p = 0.06, Figure 4E). No differences were observed in the use of inhaled corticosteroid therapy, age (data not shown) and the levels of HDAC2 protein (Figure 4F).

## Discussion

Severe asthma is characterized clinically by total or partial loss of corticosteroid sensitivity for the control of asthma symptoms [22]. Even at cellular level, macrophages [8], epithelial cells [23] and PBMCs [24] obtained from severe asthmatics have been reported to be corticosteroid insensitive *in vitro.* This study also confirmed corticosteroid insensitivity in severe asthma on TNFα-induced IL-8 in PBMCs. IL-8 is reported to be increased in sputum, serum, monocyte, and robust read-out/marker [25]–[27]. We established this system to determine steroid sensitivity using PBMCs from severe asthma and COPD as well as culture cells (U937 cells) in many research [28]–[30]. Furthermore, the combination of TNFα and anti-CD3/CD28 were also used to stimulate lymphocytes and monocytes together, and it resulted in 3–5 times higher induction of IL-8 production and more than 100 times less corticosteroid sensitivity than with TNFα alone. In fact, anti-CD3 and anti-CD28 have been shown to synergistically stimulate TNFα and IL-8 in T cells [31], suggesting that TNFα-produced from T lymphocyte stimulated further monocyte production of IL-8 in our samples. Under this stimulation, PBMCs also tended to be dexamethasone insensitive, and 33% of severe asthma patients were totally dexamethasone resistant but only 10% of healthy volunteers and 10% of non-severe asthma group were dexamethasone resistant. Furthermore, it was found that the reduction in corticosteroid responsiveness correlated with a decrease in lung function, suggesting that patients with less corticosteroid sensitivity in PBMCs *ex vivo* displayed a more severe clinical phenotype. This reduction in lung function has been reported to be associated with a systemic increase in IL-8 and TNFα in blood serum and circulating [27] and infiltrated neutrophils in the airways [32], [33] from severe asthmatics. Neutrophilic inflammation in the lung is thought to be corticosteroid-resistant [34]. Basal IL-8, a neutrophil chemoattractant, has been shown to be increased in BAL and sputum from severe asthmatics [32], [33] although release from IL-8 from PBMCs was not. More importantly, we found that TNFα/anti-CD3/CD28-induced IL-8 production was dexamethasone-insensitive particularly in severe asthma. Prednisolone was reported not to inhibit circulating neutrophils and IL-8 in the whole blood in patients with steroid-dependent asthma [35].

Several studies have been conducted in order to identify cell signalling relevant to pathogenesis of severe asthma. p38MAPK is one of the most studied signalling kinases and various compounds are currently being tested in a number of inflammatory diseases[36]–[40]. As shown in Figure 2B, the TNFα/anti-CD3/CD28-stimulated IL-8 was inhibited by SB203580 in PBMCs, which was only inhibited at the highest concentration of dexamethasone. This shows the importance of the p38α/β pathway in the corticosteroid refractory pro-inflammatory cytokine regulation in severe asthma. The IL-8 promoter is regulated by transcription factors such as AP-1 and NF-κB [41] and p38MAPK is known to activate these transcription factors either by driving direct phosphorylation or indirectly by the phosphorylation of kinases responsible for the activation of transcription factors [17]. In contrast, dexamethasone only partially inhibited AP-1 and NF-kB inhibition [42]. In addition, p38α-dependent phosphorylation of MSK results in the phosphorylation of Histone-3 (H3) leading to the recruitment of NF-κB and the transcriptional regulation of IL-8 and other inflammatory cytokines [18]. Furthermore p38MAPK enhances the stability of pro-inflammatory cytokine mRNA by phosphorylation of an AU-rich element in mRNA [19].

However, a more essential role of SB203580 was underlined in severe asthma by the restoration of corticosteroid sensitivity by this compound, particularly in the 17 most severe asthmatics (out of 20 patients we studied). Formoterol is reported to restore steroid sensitivity in PBMCs from severe asthma [24], the efficacy of which was limited in samples from patients recruited in this study, maybe due to routine medication of combination therapy of LABA and inhaled corticosteroids. Thus, our result highlights the importance of the p38α/β pathway in the restoration of corticosteroid sensitivity as previously published [43].

Earlier studies have demonstrated that treatment with IL-2/IL-4 can mimic the corticosteroid insensitivity seen in severe asthma [5], [44]. According to results obtained in a recent study, IL-2/IL-4 treatment induced p38MAPK activation [24]. Our study showed higher basal IL-2 and IL-4 production in PBMCs from severe asthma which could result in p38MAPK activation and subsequent loss of corticosteroid sensitivity. Other studies have also revealed both cytokines to be increased in blood serum of this patient group [6] and to be associated with increased corticosteroid resistance in PBMCs [44]. Goleva *et al.* confirmed these findings in T cells where IL-2/IL-4 induced corticosteroid resistance by activating p38MAPK resulting in reduced GR nuclear translocation [45]. Similarly, our *in vitro* studies confirmed that both cytokines could induce corticosteroid insensitivity in the monocytic cell line U937 with concomitant reduction of GR nuclear translocation.

In the present study, severe asthmatics showed a tendency for a reduction of GR nuclear translocation compared to healthy volunteers, mild and moderates asthmatics. However, two distinct patterns seem to present; one showing a defect in GR nuclear translocation and another with a normal GR nuclear shuttling in agreement with previous observations by Matthews *et al.* [9]. Impaired GR nuclear localization was associated with a decrease of corticosteroid sensitivity on IL-8 inhibition in severe asthmatics (Figure 1D). In addition, severe asthma patients with lower GR nuclear translocation also showed lower lung function (Figure 1E). Interestingly, “higher responders” to SB203580 in severe asthma patients had a significantly reduced GR nuclear translocation associated with reduced dexamethasone responsiveness as compared to “low/non responders”. In fact, Irusen *et al.* demonstrated that p38MAPKα has the potential to phosphorylate GR leading to a defect of GR function [5]. Other studies have also demonstrated that another kinase, c-Jun N-terminal Kinase 1 (JNK1), and p38MAPK gamma could directly phosphorylate GR at a specific serine residue (S226) [24], [46]. We confirmed that GR was phosphorylated at serine 226 in IL-2/4 corticosteroid insensitive model and that inhibition of p38MAPKα/β partially inhibited serine 226 phosphorylation. This implies that p38MAPKα phosphorylates GR in the cytoplasm and impairs GR nuclear translocation. Actually, restoration of corticosteroid sensitivity by SB203580 was shown to correlate with a defect in GR nuclear translocation.

Thus, IL-2 and IL-4 are likely to be responsible for reduced corticosteroid responsiveness as both cytokines were increased in severe asthma and induced corticosteroid insensitivity *in vitro* via reduced GR nuclear translocation due to excessive GR serine 226 phosphorylation. More importantly, p38MAPKα/β plays a major role on corticosteroid insensitive inflammation and defective steroid receptor function via increased GR phosphorylation at serine 226, resulting in reduced affinity for corticosteroid binding and decreased ability to translocate into the nuclei.

As several p38MAPK inhibitors are now been testing in clinical trials, this information or biomarkers will be useful in order to identify severe asthmatics that will respond to the treatment. This work also confirmed a heterogeneous phenotype of severe asthma based on signalling.

## Materials and Methods

### Subjects

Ten healthy volunteers, 11 patients with mild asthma, 9 patients with moderate asthma and 20 patients with severe asthma were recruited. Asthma severities were characterized using the Global Initiative for Asthma [14] guidelines and patients characteristics is summarized in Table 1. This study was approved by the Ethics Committee of the Royal Brompton & Harefield Hospitals National Health Service Trust, and all subjects gave written informed consent.

### Isolation of PBMCs

Blood was collected in acid citrate dextrose (ACD) syringes and PBMCs were separated using the ACCUSPIN™ System-HISTOPAQUE® (Sigma, Poole, UK) following manufacturer’s instructions.

### Cell Culture of U937s

U937 (human monocytic cell lines) cells were purchased from the American Tissue Culture Center (ATCC, Teddington, UK) and maintained in continuous cell culture at 37°C, 5% CO2 in RPMI-1640 medium containing 10% FCS and 15 mM glutamine. Cells (5×10^6^) were incubated with or without human IL-2 (20 ng/ml) and IL-4 (10 ng/ml) for 48 hours in RPMI-1640 medium containing 1% FCS and 15 mM glutamine.

### Whole Cell and Nuclear Extraction

PBMCs (8−25×10^6^ cells) were stimulated in the presence/absence of dexamethasone (1 µM) (Sigma) and incubated at 37°C, 5% CO_2_ in RPMI-1640 medium (10% FCS and 15 mM glutamine) for 4 hours. Whole cell extractions were performed using the Active Motif Nuclear Extraction kit (Rixensard, Belgium) following manufacturer’s instructions. U937s that were stimulated with IL-2 and IL-4 for 48 hours, then with SB203580 for 30 minutes followed by dexamethasone treatment for 1 or 4 hours. Cells were then collected for nuclear extraction using the Active Motif Nuclear Extraction kit following manufacturer’s instructions.

### Western Blot

Proteins were separated using sodium dodecylsulphate-polyacrylamide gel electrophoresis (SDS-PAGE) and transferred into a nitrocellulose membrane using the i-Blot™ Dry Blotting System (Invitrogen, Carlsbad, CA, USA) following manufacturer’s instructions. Primary antibodies against HDAC2 (Sigma), β-actin, TBP (Abcam, Cambridge, UK), Glucocorticoid Receptor (GR, E-20, Santa Cruz Biotechnology, California, USA), anti-S226 GR (Abcam) and Lamin A/C (Santa Cruz Biotechnology) were used for protein detection. Briefly, membranes were incubated with primary antibody overnight after blocking with 5% dry skimmed milk in TBS-Tween (0.05% v/v) and then with HRP conjugated secondary antibody. Bound antibodies were visualized by the ECL system (Amersham Biosciences, Buckinghamshire, UK).

### Measure of Cytokine Release

PBMCs (1×10^6^ cells/ml) were treated with SB203580 (5 µM) (Calbiochem, Darmstadt, Germany) or formoterol (Astra Zeneca, Lund, Sweden) for 30 minutes before exposing to serial dilutions of dexamethasone (10^−11^−10^−6^ M) for 1 hour. Cells were transferred into 96-well plate coated with anti-human CD3 (10 µg/ml) and CD28 antibodies (8 µg/ml) (Becton Dickinson, Oxford, UK) and TNFα (1 ng/ml) overnight at 37°C, 5% CO2. IL-2, IL-4 and IL-8 (R&D Systems, Abingdon, UK) released into the supernatant were detected by ELISA.

U937s (0.5×10^6^ cells/ml) were incubated with or without IL-2 (20 ng/ml) and IL-4 (10 ng/ml) for 48 hours, washed in PBS and seeded in 96-well plates. Cells were treated with SB203580 (5 µM) for 30 minutes before being stimulated with serial dilutions of dexamethasone (10^−11^−10^−6^ M) for 1 hour. Cells were then transferred into a 96-well plate coated anti-human TNFα (10 ng/ml) overnight at 37°C, 5% CO_2_. IL-8 levels were quantified using ELISA.

### Immunocytochemistry of GR

PBMCs, previously incubated with/without dexamethasone (1 µM), were cytospined into slides and fixed using IntraPrep™ Reagent 1 and permeabilized with IntraPrep™ Reagent 2 (Beckman Coulter, High Wycombe, UK). The method used was adapted from Li et al. (2007) [47]. The slides were analysed by confocal microscopy with imaging analysis Leica Confocal Software Lite™ (Leica, Heidelberg, Germany).

### Detection of Phosphorylated GR

Human monocytic U937, maintained in continuous cell culture at 37°C, 5% CO_2_ in RPMI-1640 medium containing 10% foetal calf serum (FCS) and 15 mM glutamine were stimulated with IL-2 and IL-4 for 48 hours in minimal media (1% FCS) to induce corticosteroid insensitivity. Cells were then treated with SB203580 (5 µM) for 30 minutes prior whole-cell extraction and SDS-PAGE/western-blotting analysis. Phosphorylation of Serine 226 of level was determined with anti-S226 GR antibody (Abcam) and normalized to GR expression.

### Statistical Analysis

Clinical data are expressed as median and interquartile range. The effect of SB203580 on clinical data is expressed as average ± SEM. Results were analysed using Kruskal Wallis and comparisons were made by Mann-Whitney using the Graph Pad Prism Software (Prism, San Diego, CA). Correlation was analysed by non-parametric Spearman method. Comparisons of two values were analysed with Wilcoxon matched pair test or paired t-test. The in vitro results were expressed as average ± SEM. P<0.05 was considered statistically significant.

## Supporting Information

Figure S1 **A. PBMCs from healthy volunteers (HV) (n = 10), non-severe asthmatics (NSAA) (n = 20) and severe asthmatics (SA) (n = 20) were incubated 1 hour with Dex (10^−11^−10^–6^M) followed by 24 hours with anti-CD3/28 plus TNFα. IC_50_dex was measured for IL-8 in all patients.** Some patients became completely resistant to Dex and their IC_50_dexs could not be calculated. They were given a nominal value of 10^−4^ M. Data was plotted as median ± SEM. **B.** PBMCs from HV (n = 10), NSA (n = 20) and SA (n = 20) were seeded in 96-well plates and IL-2 cytokine release was measured using ELISA. **C.** PBMCs from HV (n = 10), NSA (n = 20) and SA (n = 20) were seeded in 96-well plates and IL-4 cytokine release was measured using ELISA. **D.** PBMCs were incubated with/without Dex (1 µM) for 4 hours. GNI was measured by immunocytochemistry in HV (n = 9), NSA (n = 14) and SA (n = 19). **E.** HDAC2 protein expression was determined by SDS-PAGE/Western blotting and normalized using the expression of β-actin in HV (n = 8), NSA (n = 20) and SA (n = 18). A representation blot showing results from four patients is shown. Only ‘N’ samples are shown in the graph. (N =  non-treatment, D = dex (1 µM)).
(TIF)

Figure S2 **A. Add-on treatments in severe asthma. A.** PBMCs from severe asthmatics were treated with formoterol (1 nM) or SB203580 (5 µM) for 30 minutes followed by 1 hour stimulation with Dex (10^−11^–10^−6^ M) and 24 hour with anti-CD3/28 plus TNFα. IL-8 release was measured by ELISA and IC_50_dexs calculated. The improvement on corticosteroid sensitivity was assessed for each add-on treatment by calculating the ratio (fold) change of IC_50_dex before and after treatment. A “heat-map” was constructed using the ratio for each svere asthmatic. **B.** PBMCs from severe asthmatics were treated with formoterol (1 nM) for 30 minutes followed by 24 hour with anti-CD3/28 plus TNFα. IL-8 release was assessed using ELISA.
(TIF)
