# Supplementary figures and images for: Restoration of Corticosteroid Sensitivity by p38 Mitogen Activated Protein Kinase Inhibition in Peripheral Blood Mononuclear Cells from Severe Asthma
Source: PLoS One. 2012 Jul 23;7(7):e41582. doi: 10.1371/journal.pone.0041582 (PMC3402424; doi:10.1371/journal.pone.0041582)

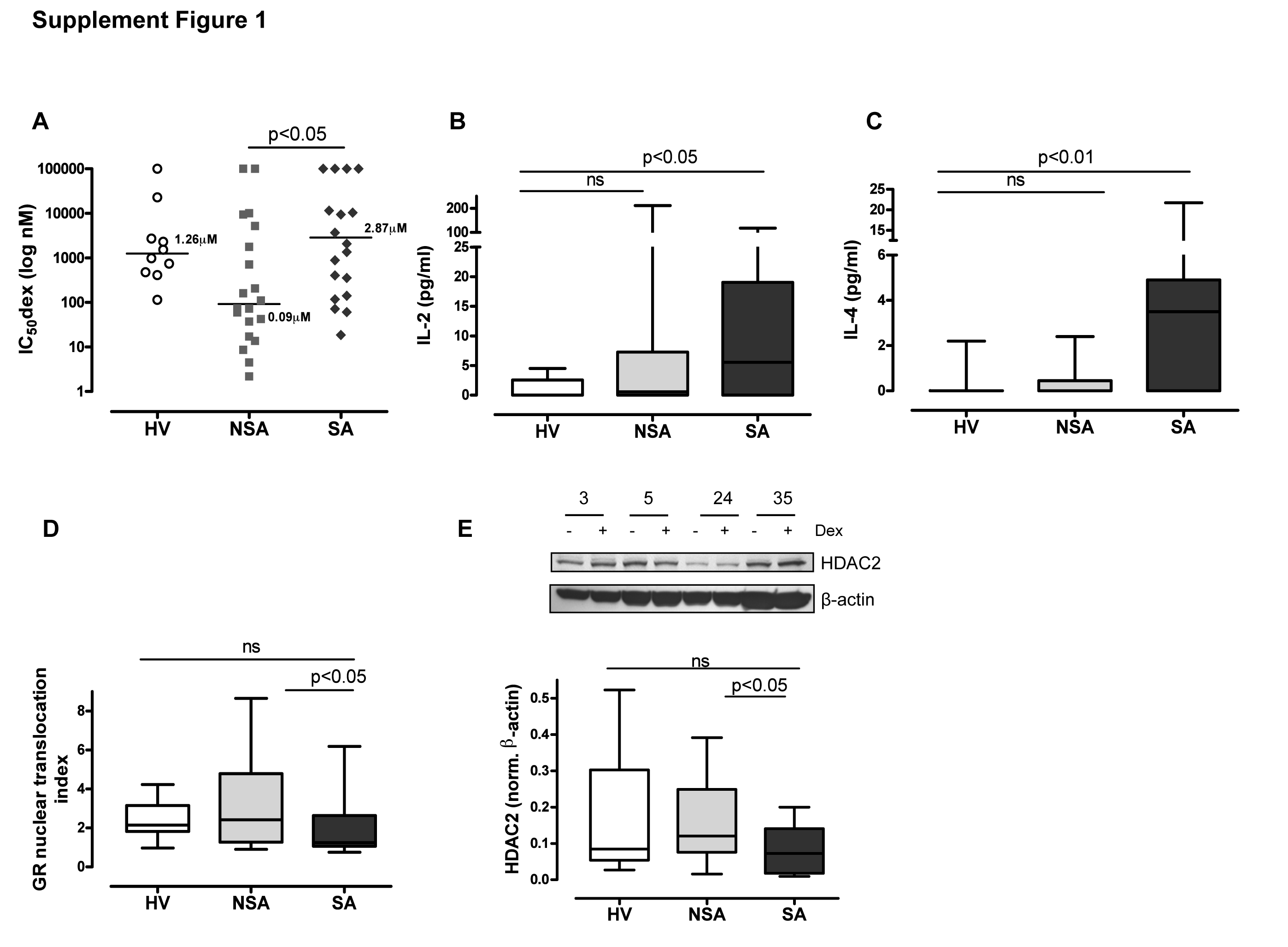

Supplement: Figure S1 — A. PBMCs from healthy volunteers (HV) (n = 10), non-severe asthmatics (NSAA) (n = 20) and severe asthmatics (SA) (n = 20) were incubated 1 hour with Dex (10−11−10–6M) followed by 24 hours with anti-CD3/28 plus TNFα. IC50dex was measured for IL-8 in all patients. Some patients became completely resistant to Dex and their IC50dexs could not be calculated. They were given a nominal value of 10−4 M. Data was plotted as median ± SEM. B. PBMCs from HV (n = 10), NSA (n = 20) and SA (n = 20) were seeded in 96-well plates and IL-2 cytokine release was measured using ELISA. C. PBMCs from HV (n = 10), NSA (n = 20) and SA (n = 20) were seeded in 96-well plates and IL-4 cytokine release was measured using ELISA. D. PBMCs were incubated with/without Dex (1 µM) for 4 hours. GNI was measured by immunocytochemistry in HV (n = 9), NSA (n = 14) and SA (n = 19). E. HDAC2 protein expression was determined by SDS-PAGE/Western blotting and normalized using the expression of β-actin in HV (n = 8), NSA (n = 20) and SA (n = 18). A representation blot showing results from four patients is shown. Only ‘N’ samples are shown in the graph. (N = non-treatment, D = dex (1 µM)). (TIF) [file pone.0041582.s001.tif]

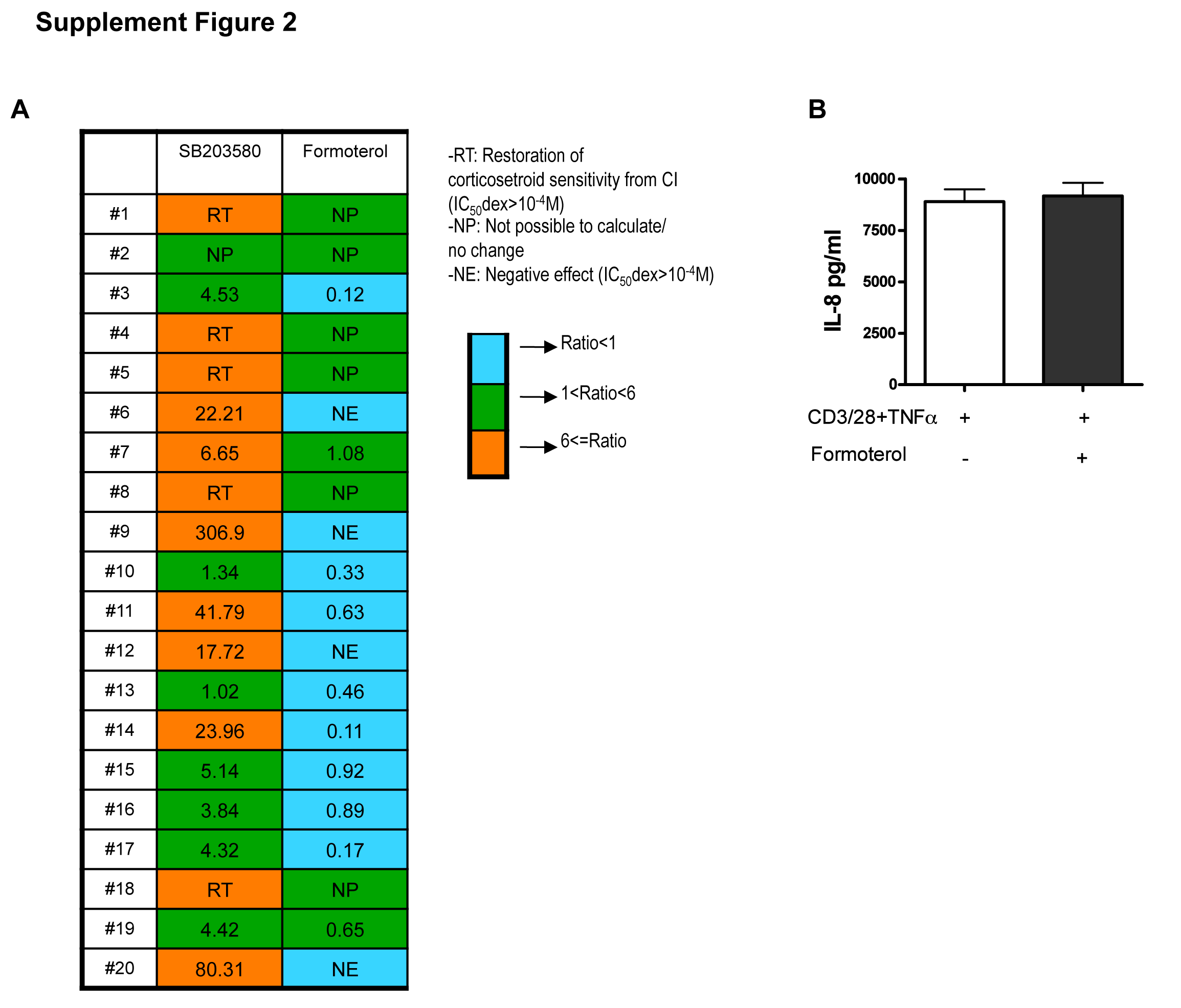

Supplement: Figure S2 — A. Add-on treatments in severe asthma. A. PBMCs from severe asthmatics were treated with formoterol (1 nM) or SB203580 (5 µM) for 30 minutes followed by 1 hour stimulation with Dex (10−11–10−6 M) and 24 hour with anti-CD3/28 plus TNFα. IL-8 release was measured by ELISA and IC50dexs calculated. The improvement on corticosteroid sensitivity was assessed for each add-on treatment by calculating the ratio (fold) change of IC50dex before and after treatment. A “heat-map” was constructed using the ratio for each svere asthmatic. B. PBMCs from severe asthmatics were treated with formoterol (1 nM) for 30 minutes followed by 24 hour with anti-CD3/28 plus TNFα. IL-8 release was assessed using ELISA. (TIF) [file pone.0041582.s002.tif]
